# Age-specific prevalence, subtypes and risk factors of metabolic diseases in Chinese adults and the different patterns from other racial/ethnic populations

**DOI:** 10.1186/s12889-022-14555-1

**Published:** 2022-11-14

**Authors:** Qiuyu Cao, Ruizhi Zheng, Ruixin He, Tiange Wang, Min Xu, Jieli Lu, Meng Dai, Di Zhang, Yuhong Chen, Zhiyun Zhao, Shuangyuan Wang, Hong Lin, Weiqing Wang, Guang Ning, Yufang Bi, Yu Xu, Mian Li

**Affiliations:** 1 Department of Endocrine and Metabolic Diseases, Shanghai Institute of Endocrine and Metabolic Diseases, Ruijin Hospital, Shanghai Jiao Tong University School of Medicine, 197 Rui-Jin 2nd Road, Shanghai, 200025 China; 2 Shanghai National Clinical Research Center for metabolic Diseases, Key Laboratory for Endocrine and Metabolic Diseases of the National Health Commission of the PR China, Shanghai Key Laboratory for Endocrine Tumor, State Key Laboratory of Medical Genomics, Ruijin Hospital, Shanghai Jiao Tong University School of Medicine, 197 Rui-Jin 2nd Road, Shanghai, 200025 China

**Keywords:** Metabolic disease, Age, Ethnicity, Risk factor

## Abstract

**Background:**

Age has substantial influence on metabolic diseases patterns. Ethnic disparities of metabolic characteristics between Chinese and other populations also exist. Large-scale investigations of age-specific prevalence, subtypes and modifiable risk factors of metabolic disorders are essential to promote individualized strategies for the control and prevention of metabolic diseases in multi-ethnic populations. The study aims to address the age-specific prevalence, subtype characteristics and risk factor profiles of metabolic diseases among different races/ethnicities.

**Methods:**

We analyzed data from the China Noncommunicable Disease Surveillance 2010 and the National Health and Nutrition Evaluation Survey (NHANES). We examined the prevalence and subtypes of hypertension, diabetes and hyperlipidemia across age groups in four ethnic populations. We also investigated the odds ratios (ORs) of metabolic diseases associated with 11 classical risk factors in the young and the elder Mainland Chinese.

**Results:**

The sex and BMI standardized prevalence of hypertension in Chinese aged 18–40 years was 18.5% and was the highest among the four populations. The main pathophysiologic subtype of diabetes was characterized by insulin resistance, instead of β-cell dysfunction in Mainland Chinese, and this pattern was more evident in obese subjects. The major subtype of hyperlipidemia in Mainland Chinese was hypertriglyceridemia, while Non-Hispanic Whites and Blacks were more prone to high low-density lipoprotein cholesterol. For risk of hypertension, diabetes and hyperlipidemia, young Chinese adults were more prone to general and central obesity than older ones. The other factors showed similar effects on the young and the old.

**Conclusions:**

The age-specific prevalence, subtypes and risk factors of metabolic diseases were substantially different in Chinese and other ethnic/racial populations.

**Supplementary Information:**

The online version contains supplementary material available at 10.1186/s12889-022-14555-1.

## Introduction

Cardiovascular diseases (CVD) have become a major cause of death and disability worldwide, and metabolic disorders such as hypertension, diabetes and hyperlipidemia significantly increase the risk of cardiovascular diseases [1]. The metabolic health status of Chinese adults has been deteriorating in the past decades, which led to heavy burden of cardiovascular disease in China [2].

Age is a dominant risk factor of metabolic diseases. Nevertheless, the prevalence of metabolic disorders including hypertension, diabetes and obesity has been increasing substantially in young adults over the past decades [3–5]. Therefore, it is of great importance to estimate the prevalence of metabolic diseases across adults of all ages. Of note, the characteristics or subtype of metabolic disorders are diverse between the old and the young. The previous evidence of age-specific characteristics of metabolic diseases was primarily derived from western population, for instance, participants of the National Health and Nutrition Evaluation Survey (NHANES) [6] and the Whitehall II cohort study [7]. And data of metabolic diseases subtype characteristics in Mainland Chinese is quite scarce, particularly in young subjects aged 18–40 years. Considering the serious epidemic of metabolic diseases in Chinese young adults, investigation of age-specific metabolic diseases subtype in adults across life span could promote evaluation of the current burden of metabolic disorders in China and guidance of individualized management targets in people of all ages.

In addition, among different ethnic/racial populations, the discrepancy of metabolic diseases subtype patterns is evident [8, 9]. It was reported that compared with non-Hispanic Whites, Asian Americans and Mexican Americans had an increased prevalence of high triglycerides (TG), while the prevalence of high low-density lipoprotein cholesterol (LDL-C) was increased among Asian Indians and Japanese [10]. Therefore, it is quite essential to further analyze the age-specific characteristics of metabolic diseases subtypes among different ethnic/racial populations, which could provide novel evidence to specific management strategies for metabolic disorders in Mainland Chinese and other ethnic/racial populations.

Furthermore, the age-specific profiles of metabolic diseases risk factors require further investigation. Previous studies supported that the early-onset or young-onset hypertension or diabetes in subjects younger than 40 years might have different pathologic mechanisms or risk factor profiles with the late-onset diseases. We have demonstrated in our previous nationwide study that the age disparities of modifiable risk factor patterns for cardiovascular disease, all-cause mortality and diabetes were significant and highlighted the significance of estimating age-specific risk factors profiles for metabolic diseases to propose individualized prevention guidelines [11, 12]. However, data from young adults aged 18–40 years was quite insufficient in previous studies, leading to a lack of clinical recommendations for the young metabolic disease patients in current guidelines [13, 14].

Herein, to address the age-specific characteristics in metabolic diseases prevalence, subtypes and risk factors in multi-ethnic/racial populations, we used data from a nationally representative sample of 98,658 Chinese adults aged over 18 years and data from the NHANES.

## Methods

An extended methods section is available in Additional file 1.

### Study population

The data of Chinese adults in this study were collected through the China Noncommunicable Disease Surveillance 2010. It included all 162 study sites from the Chinese Center for Disease Control and Prevention (CDC) National Disease Surveillance Point System, which covered the major geographic areas of all 31 provinces, autonomous regions, and municipalities in mainland China. Details on study design and protocol had been described previously [15].

The NHANES is a cross-sectional, nationally representative survey conducted by the National Center for Health Statistics (NCHS) of the Centers for Disease Control in the United States (U.S.). Details of the study protocols and study design are available in public (http://www.cdc.gov/nchs/nhanes.htm).

For the current analysis, data of 98,658 participants from the China Noncommunicable Disease Surveillance 2010 and 28,132 individuals from NHANES 2005–2016 in races of Mexican American, Non-Hispanic White and Non-Hispanic Black was included. The Flow Chart of study participants was presented in Additional file 1: Fig. S1. All the Mainland Chinese participants provided written informed consent and the Ethical Review Committee of the Chinese Center for Disease Control and Prevention and other participating institutes approved the study protocol, with ethics approval ID: 201010. The NCHS ethics review board reviewed and approved the NHANES survey and participants provided informed consent.

### Data collection

Data collection of Mainland Chinese participants was conducted in examination centers at local health stations or community clinics by trained staff according to a standard protocol. Body weight, height and waist circumstance (WC) were measured, and body mass index (BMI) was calculated as weight (kg) divided by squared height (m^2^). Details on the measurements of blood pressure, fasting plasma glucose (FPG), 2 h-postload glucose (2 h-PPG), glycated hemoglobin (HbA1c) and blood lipids are explained elsewhere [15]. The details of physical measures and laboratory tests in NHANES are described online (https://www.cdc.gov/nchs/nhanes). The homoeostasis model assessment of insulin resistance (HOMA-IR) index was calculated with the following formula: [16] fasting insulin (μIU/mL) × fasting glucose (mg/dL)/405 and insulin resistance (IR) was defined as HOMA-IR within the highest quartile. The homoeostasis model assessment of β-cell function (HOMA-B) index was calculated as follows: (360 × fasting insulin [μIU/mL])/ (fasting glucose [mg/dl]-63), and β-cell dysfunction was defined as HOMA-B within the lowest quartile.

A questionnaire including demographic data, lifestyle, chronic diseases history and medication usage was administered by trained staff in China Noncommunicable Disease Surveillance 2010. The NHANES survey also conducted a questionnaire interview, collecting similar data.

### Definitions of metabolic diseases and subtypes

Hypertension was defined according to the 2018 European Society of Cardiology (ESC)/ European Society of Hypertension (ESH) guideline for the management of hypertension, [13] and was categorized as Isolated Systolic Hypertension (ISH, only systolic blood pressure (SBP) ≥ 140 mmHg), Isolated Diastolic Hypertension (IDH, only diastolic blood pressure (DBP) ≥ 90 mmHg) and Systolic Diastolic Hypertension (SDH, SBP ≥ 140 mmHg and DBP ≥ 90 mmHg). Diabetes was ascertained according to the American Diabetes Association (ADA) 2021 criteria: [17] FPG 126 mg/dL (7·0 mmol/L) or higher, 2 h-PPG 200 mg/dL (11·1 mmol/L) or higher, or HbA1c 6·5% or higher, or a self-reported diagnosis of diabetes by health-care professionals. According to different pathophysiology, diabetes was defined as diabetes only with IR, only with β-cell dysfunction, with both conditions and without either condition. Diabetes was also divided to 7 groups according to the number and type of elevated glycemic measurements. Hyperlipidemia was defined as plasma TG ≥ 200 mg/dl or LDL-C ≥ 160 mg/dl or taking lipid-lowering medications and was categorized as hypertriglyceridemia, high LDL-C and both disorders.

### Statistical analysis

Statistical analyses were performed using SAS system, version 9.4 (SAS Institute Inc) and R version 3.6.1. The appropriate weights and design factors were invoked in all the analyses to account for the multistage probability sampling design of the two surveys. For analyses using NHANES data, we calculated the comprehensive weight by dividing the sampling weight in each survey cycle by 6, and invoked it in all the analyses. Baseline characteristics of study participants were described in means (95% confidence interval (CI)) for continuous variables and percentages (95%CI) for categorical variables by race and age. The sex and BMI standardized metabolic diseases prevalence of participants was presented in percentages (95%CI) by race, age and medication status. The generalized additive model with penalized smoothing splines was used to delineate the dose-response associations of age with each cardiometabolic metrics in different racial subjects.

To visibly compare the age and ethnic differences in the subtypes of metabolic diseases, we depicted the distribution of each hypertensive, diabetes and hyperlipidemic subtype by age in 4 ethnic groups for untreated individuals by stacked bar chart. And we further analyzed the distribution of diabetes subtypes in obese and non-obese Mainland Chinese. To analyze metabolic diseases risk attributable to 11 modifiable risk factors (including general obesity, central obesity, lower education level, lower family income, lower occupation level, low grade marital status, current smoking, current drinking, physical inactivity, unhealthy diet and exposure to high PM2.5 level) in Mainland Chinese, we used 3 adjusted univariable logistic regression models to identify the age-specific odds ratios and 95%CI for each risk factor for metabolic diseases. The interaction term between each risk factor and age group (1 for 18–40 years, and 0 for over 40 years) was added separately to the logistics model containing age, the risk factor and other covariates, and the P for interaction term and young-to-old ratio of odds ratio for each risk factor could be obtained [18]. We also performed sensitivity analyses in the population including subjects on medication and the results were shown in Additional file 1: Fig. S5, Fig. S6 and Table S2.

## Results

### Age and race-specific metabolic profiles and metabolic diseases prevalence

The baseline characteristics of participants are presented by race and age in Additional file 1: Table S1. The adults younger than 40 years had lower BMI, WC, blood pressure, blood glucose and blood lipids than those older than 40 years. The Chinese adults had lower BMI, WC, FPG, total cholesterol and LDL-C, but higher blood pressure than the other ethnic subjects. The prevalence of metabolic diseases in people of different race and age is shown in Table 1. The prevalence of metabolic diseases rose with age. In Chinese young adults aged 18–40 years, the sex and BMI standardized prevalence of hypertension was 18.5% and was the highest among the four populations. Notably, hypertension on medication (1.2%) was far less frequent than hypertension not on medication (16.9%) in Chinese young adults, and the prevalence was much lower than those of people of other races/ethnicities. Besides, the prevalence of diabetes and hyperlipidemia of Chinese young adults was 6.2 and 11.6%.

**Table 1:** Metabolic diseases prevalence in people of different ethnic and age

Prevalence by age (95%CI)	Mainland Chinese		Mexican American		Non-Hispanic White		Non-Hispanic Black	
Hypertension	**On medication**	**Not on medication**	**On medication**	**Not on medication**	**On medication**	**Not on medication**	**On medication**	**Not on medication**
18 ~ 40 years	1.6 (1.3–1.9)	16.9 (15.9–17.9)	3.5 (2.4–4.6)	5.4 (4.1–6.7)	4.3 (3.6–5.0)	7.3 (6.3–8.3)	6.8 (5.6–8.0)	7.9 (8.5–9.3)
41 ~ 50 years	6.2 (5.7–6.7)	27.4 (26.3–28.5)	8.8 (6.4–11.2)	7.3 (4.9–9.7)	13.8 (11.8–15.8)	10.4 (8.5–12.2)	25.6 (22.3–28.9)	13.9 (11.0–16.8)
51 ~ 60 years	14.2 (13.3–15.1)	35.4 (34.0–36.8)	23.5 (18.6–28.4)	9.0 (6.2–11.8)	25.4 (22.8–28.0)	11.8 (9.7–13.8)	42.1 (38.2–46.0)	15.8 (12.7–18.9)
61 ~ 70 years	22.1 (20.9–23.3)	41.9 (40.3–43.5)	40.0 (34.7–45.7)	18.8 (14.3–23.3)	39.1 (35.9–42.3)	13.6 (11.2–16.0)	62.5 (58.5–66.3)	12.9 (10.2–15.6)
> 70 years	26.1 (24.3–27.3)	47.1 (45.0–49.2)	55.3 (49.3–61.3)	17.3 (12.6–22.0)	56.9 (54.6–59.2)	14.9 (13.3–16.5)	72.6 (69.0–76.2)	12.0 (8.9–15.1)
Diabetes	**On medication**	**Not on medication**	**On medication**	**Not on medication**	**On medication**	**Not on medication**	**On medication**	**Not on medication**
18 ~ 40 years	1.0 (0.8–1.2)	5.2 (4.7–5.7)	2.2 (1.0–3.4)	3.3 (2.2–4.4)	2.6 (1.7–3.5)	1.9 (1.2–2.6)	3.3 (2.1–4.5)	2.7 (1.6–3.8)
41 ~ 50 years	2.6 (2.3–2.9)	8.5 (7.8–9.2)	11.0 (7.0–15.0)	11.5 (7.4–15.6)	4.4 (3.0–5.8)	3.9 (2.7–5.1)	9.7 (6.7–12.7)	8.5 (5.6–11.4)
51 ~ 60 years	4.9 (4.4–5.4)	12.6 (11.8–13.4)	19.3 (13.9–24.7)	10.4 (7.4–13.4)	8.8 (6.7–10.9)	6.6 (4.6–8.6)	20.1 (16.2–24.0)	8.3 (5.5–11.1)
61 ~ 70 years	7.4 (6.7–8.1)	15.1 (13.9–16.3)	31.7 (26.0–37.2)	16.2 (11.4–21.0)	11.9 (9.7–14.1)	8.3 (6.1–10.5)	27.0 (22.9–31.1)	11.2 (8.5–13.9)
> 70 years	7.2 (6.1–8.3)	16.9 (15.4–18.4)	34.8 (27.8–41.8)	19.8 (14.4–24.2)	18.5 (16.3–20.7)	14.2 (12.3–16.1)	34.7 (29.5–39.9)	16.3 (12.1–20.5)
Hyperlipidemia	**On medication**	**Not on medication**	**On medication**	**Not on medication**	**On medication**	**Not on medication**	**On medication**	**Not on medication**
18 ~ 40 years	0.3 (0.2–0.4)	11.3 (10.6–12.0)	0.1 (0.0–0.2)	14.0 (11.7–16.6)	0.2 (0.0–0.4)	12.1 (10.4–13.8)	0.2 (0.1–0.3)	7.5 (5.7–9.3)
41 ~ 50 years	0.8 (0.6–1.0)	14.7 (14.0–15.4)	0.6 (0.2–1.0)	16.3 (12.1–20.5)	1.8 (1.1–2.5)	16.9 (13.5–20.3)	0.8 (0.4–1.2)	11.9 (9.2–14.6)
51 ~ 60 years	1.2 (1.0–1.4)	14.8 (13.9–15.7)	5.5 (2.7–8.3)	25.3 (19.1–31.5)	3.2 (2.0–4.4)	14.3 (11.5–17.1)	2.9 (1.2–4.6)	11.0 (7.4–14.6)
61 ~ 70 years	1.3 (1.0–1.6)	13.5 (12.4–14.6)	4.1 (1.8–6.4)	10.7 (6.9–14.5)	4.6 (2.9–6.3)	8.0 (5.9–10.1)	3.2 (1.6–4.8)	8.2 (5.4–11.0)
> 70 years	0.8 (0.5–1.1)	12.3 (11.0–13.6)	3.2 (1.5–4.9)	8.4(4.3–12.5)	4.8 (3.5–6.1)	8.3 (6.9–9.7)	1.1 (0.3–1.9)	3.8 (2.0–5.6)

*CI* Confidence interval

**Fig. 1:**
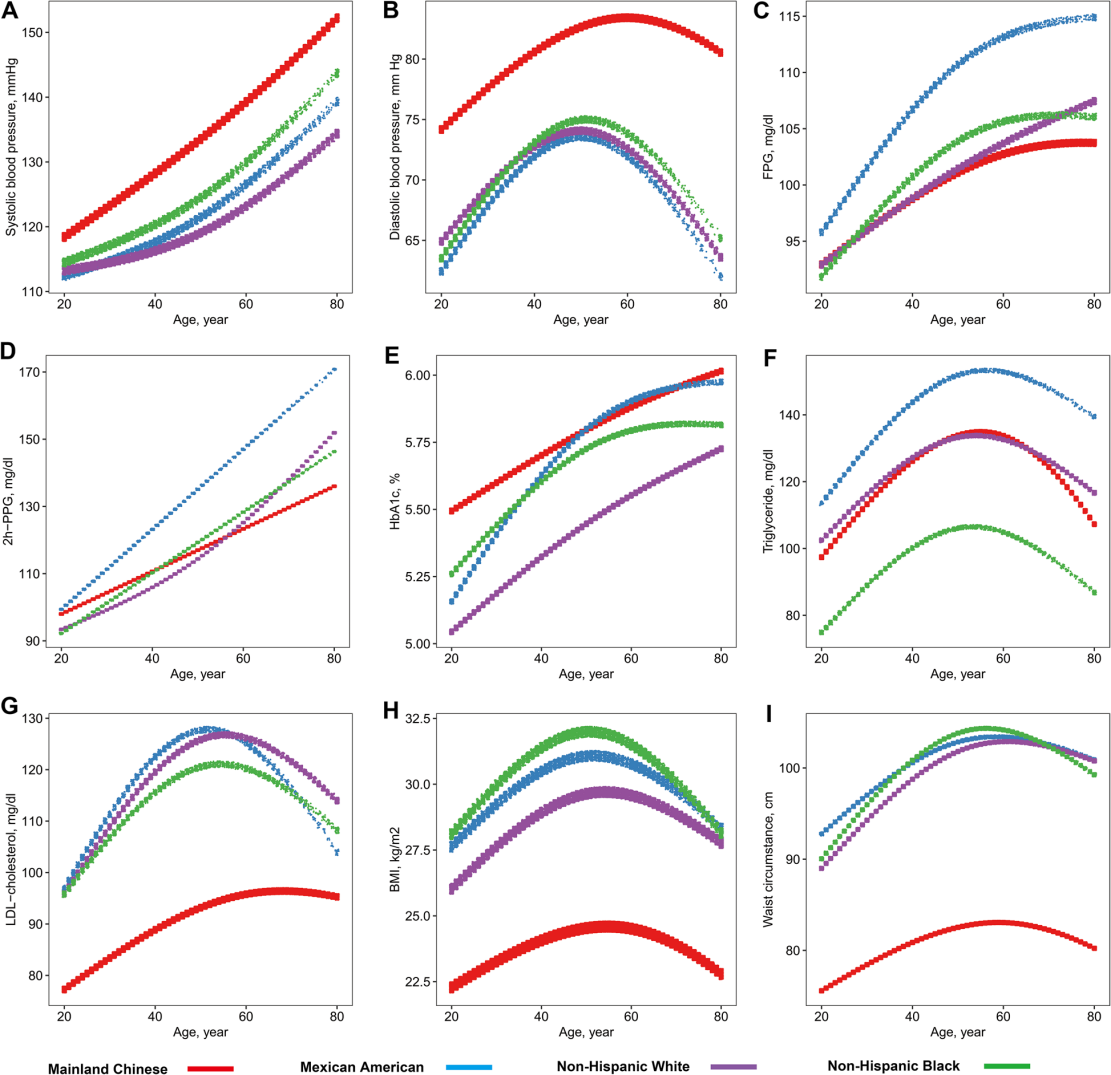
Association of age with cardiometabolic metrics in different racial/ethnic adults. (**A**) systolic blood pressure, (**B**) diastolic blood pressure, (**C**) fasting plasma glucose, (**D**) 2 h-postload plasma glucose, (**E**) glycated hemoglobin, (**F**) triglycerides, (**G**) low-density lipoprotein cholesterol, (**H**) Body mass index, (**I**) Waist circumstance. (Red curves indicate Chinese, blue curves indicate Mexican American, purple curves indicate Non-Hispanic Whites, green curves indicate Non-Hispanic Black). For the models with dependent variables of SBP and DBP, the subjects are excluded for administration of anti-hypertensive medicine. For the models with dependent variables of TG and LDL-C, the subjects are excluded for administration of lipid lowering medicine. For the models with dependent variables of FPG, 2 h-PPG and HbA1c, the subjects are excluded for administration of anti-diabetic medicine

The dose-response associations between age and metabolic metrics are shown by ethnicity in Fig. 1. Among all the racial subjects, age presented positive associations with SBP, FPG, 2 h-PPG and HbA1c, and showed curvilinear associations with DBP, TG, LDL-C, BMI and WC, which were the highest among adults aged around 50 years.

### Age and race-specific metabolic diseases subtypes

**Fig. 2:**
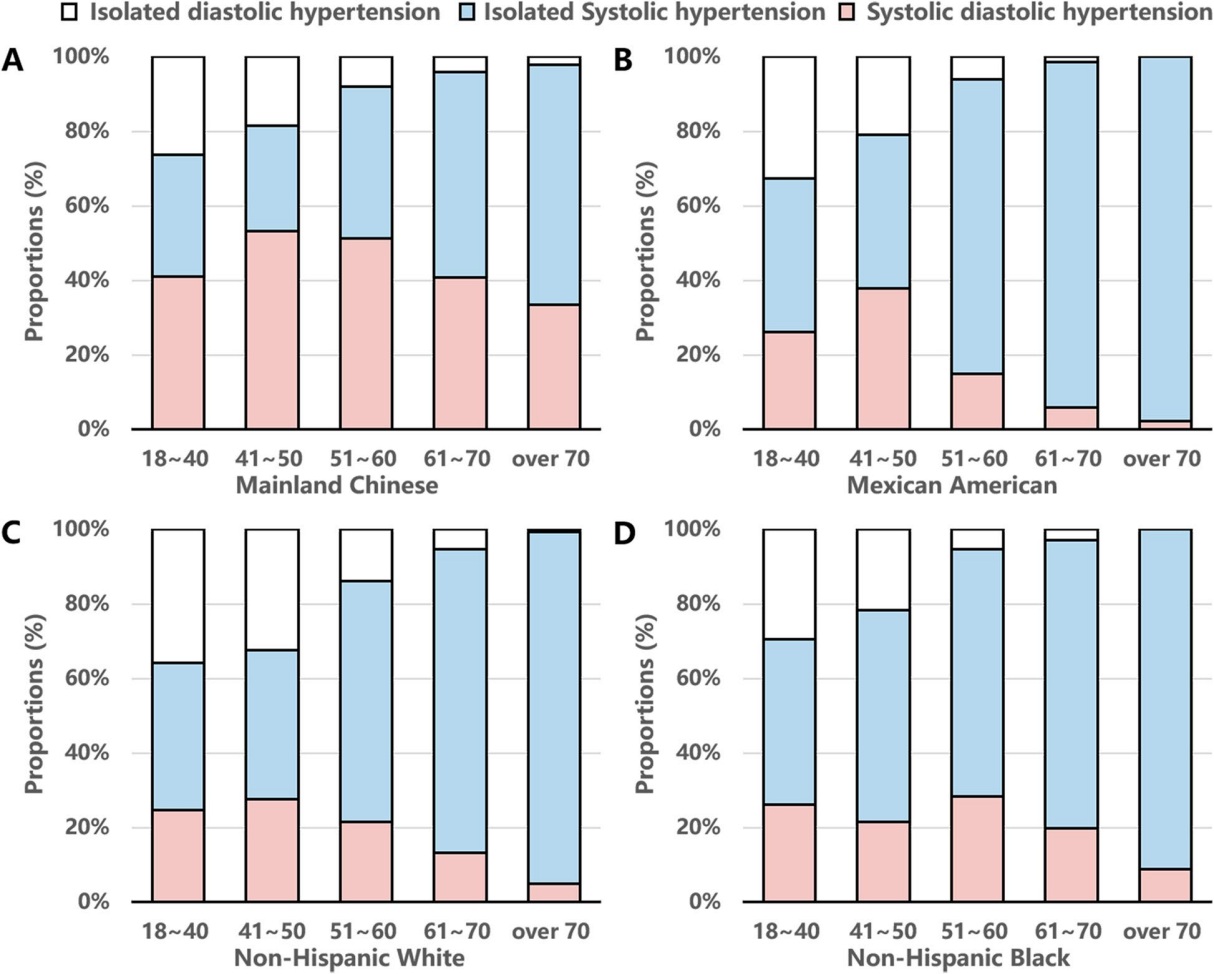
Frequency distribution of untreated hypertensive individuals by age and hypertension subtypes. **A** Mainland Chinese, **B** Mexican American, **C** Non-Hispanic White, **D** Non-Hispanic Black

The distribution of untreated hypertensive individuals according to hypertension subtypes by age and race was shown in Fig. 2. The proportion of ISH increased with age in all ethnic subjects, and that of IDH decreased with age. The proportion of SDH in Chinese untreated hypertensive adults was significantly higher than that in other ethnic subjects.

Untreated patients with diabetes were divided by characteristics of insulin resistance and β-cell dysfunction in Additional file 1: Fig. S2. Most of the Chinese untreated patients with diabetes had insulin resistance. To address the influence of obesity on diabetes pathophysiology, the Chinese patients with diabetes were further divided by BMI in Additional file 1: Fig. S3. Among non-obese patients, diabetes with β-cell dysfunction dominated, whilst among obese subjects, diabetes with insulin resistance took the majority. Notably, compared with Mainland Chinese, other ethnic/racial subjects had a decreased prevalence of β-cell dysfunction and most patients with diabetes were characterized by insulin resistance. Untreated patients with diabetes were also separated by elevated glycemic metrics, namely FPG, 2 h-PPG and HbA1c, in Additional file 1: Fig. S4. The proportions of diabetes diagnosed only by FPG, 2 h-PPG or HbA1c were similar in Chinese adults with diabetes.

**Fig. 3:**
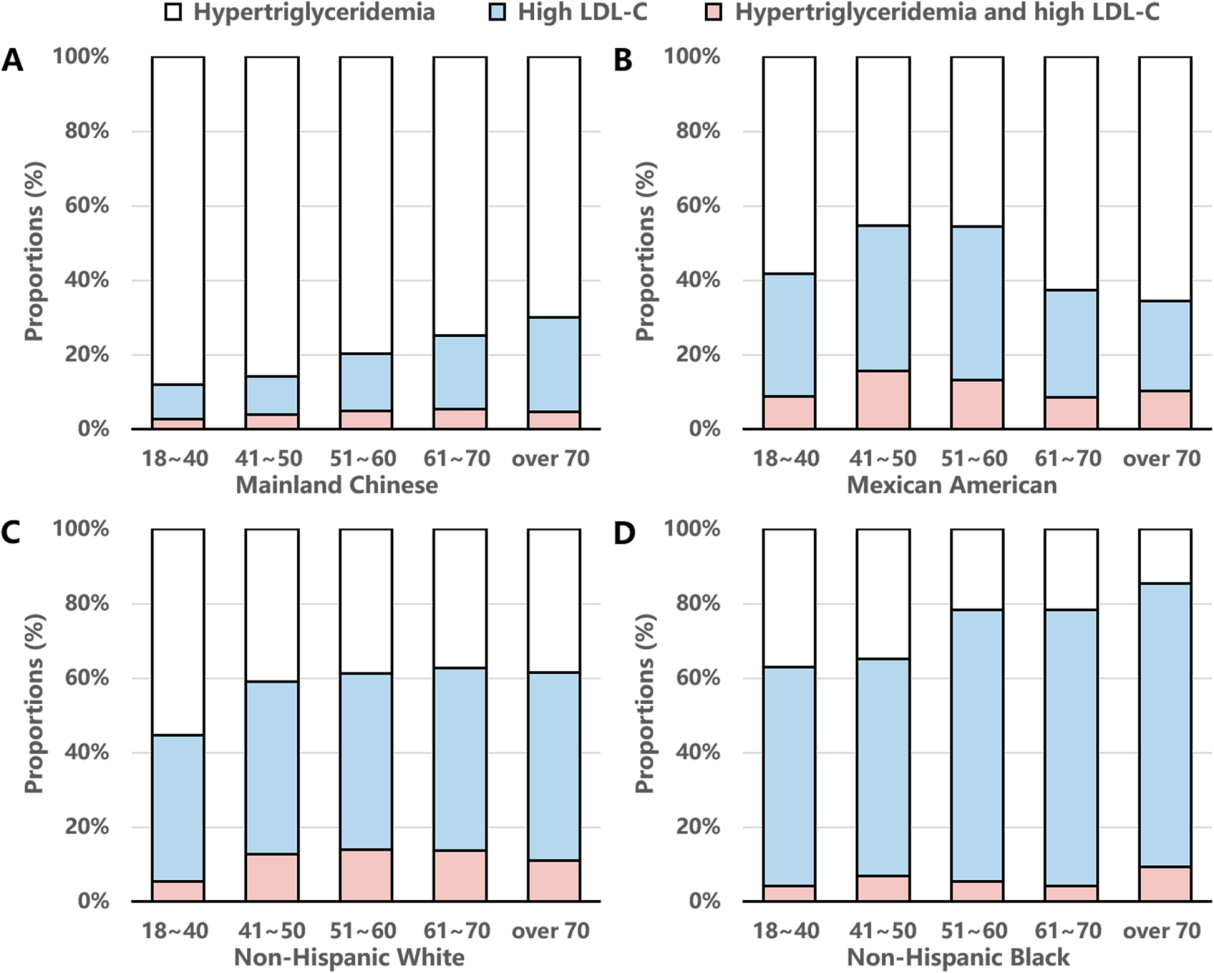
Frequency distribution of untreated hyperlipidemia individuals by age and hyperlipidemia subtypes. **A** Mainland Chinese, **B** Mexican American, **C** Non-Hispanic White, **D** Non-Hispanic Black. LDL-C, low density lipoprotein cholesterol

The distribution of untreated hyperlipidemic individuals by age, race and hyperlipidemia subtypes was demonstrated in Fig. 3. In Chinese and Mexican American hyperlipidemic patients, hypertriglyceridemic adults took the majority, whereas in Non-Hispanic White and Black individuals, high LDL-C patients dominated.

### Age-specific metabolic risk factor profiles in mainland Chinese

**Fig. 4:**
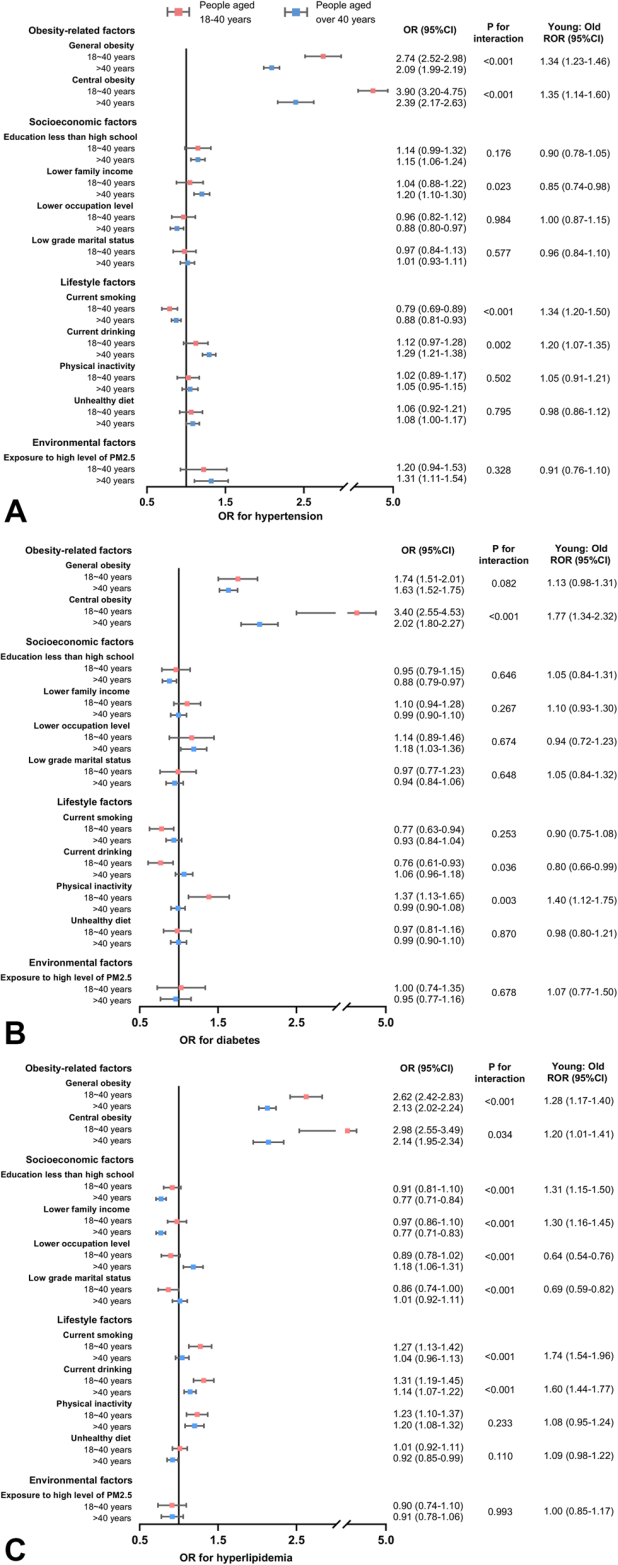
Age-specific odds ratios and young-to-old ratio of odds ratios for risk factors and metabolic diseases. **A** OR for hypertension, **B** OR for diabetes, **C** OR for hyperlipidemia. OR, odds ratio; CI, confidence interval; ROR, ratio of odds ratio. Pink squares represent odds ratios for people aged 18–40 years, and blue squares represent odds ratios for people aged over 40 years, horizontal lines indicate corresponding 95% confidence intervals around odds ratios. All models were adjusted for age and sex. In addition, the model used for hypertensive risk factors analysis was adjusted for hypertension family history and diagnosis of diabetes and hyperlipidemia. The model used for diabetes risk profiles analysis was additionally adjusted for diabetes family history and diagnosis of hypertension and hyperlipidemia. The model used for hyperlipidemic risk profiles analysis was further adjusted for diagnosis of hypertension and diabetes

In Fig. 4, the risk of hypertension, diabetes and hyperlipidemia attributable to 11 classical metabolic risk factors in Chinese individuals with different age was presented. Firstly, the risk of 3 metabolic diseases associated with obesity and central obesity was remarkably higher in young adults < 40 years than in the elder. The ORs (95%CI) for hypertension, diabetes and hyperlipidemia attributable to general obesity were2.74 (2.52–2.98), 1.74 (1.51–2.01) and 2.62 (2.42–2.83) in the young, and 2.09 (1.99–2.19), 1.63 (1.52–1.75), and 2.13 (2.02–2.24) in the old, with each P for interaction < 0.05, except for diabetes. Similarly, the ORs (95%CI) for hypertension, diabetes and hyperlipidemia attributable to central obesity were 3.90 (3.20–4.75), 3.40 (2.55–4.53) and 2.98 (2.55–3.49) in the young, and 2.39 (2.17–2.63), 2.02 (1.80–2.27), and 2.14 (1.95–2.34) in the old, with each P for interaction < 0.05. Besides, physical inactivity showed a stronger association with risk of diabetes in adults < 40 years. But the other social, lifestyle and environmental factors showed similar effects on the young and the old. The results including people taking anti-hypertensive, anti-diabetic and lipid lowering medication remained similar in sensitivity analysis.

## Discussion

In this cross-sectional study of two nationwide surveys in China and United States including subjects aged over 18 years, we analyzed the age-specific prevalence, subtypes and risk factors of metabolic diseases in multi-ethnic populations. Although compared with people aged over 40 years, the prevalence of metabolic disorders was lower in young adults aged 18–40 years, 18.5, 6.2 and 11.6% of them were detected as hypertension, diabetes or hyperlipidemia in China. Furthermore, the metabolic diseases subtype and risk factor profiles of young adults showed obvious difference from those of the midlife and old individuals. In addition, we revealed ethnic/racial disparities in metabolic diseases subtype patterns. These results could promote more specific interpretation and adoption of clinical guidelines in populations with different age and ethnicity/race.

Age has a great impact on metabolic parameters, but the trajectories of different metrics are not quite the same [19]. We observed that SBP and blood glycemic measurements increased linearly with age, while DBP, TG, LDL-C, BMI and WC reached high levels around 50 years. These results were consistent with previous findings from longitudinal studies [19, 20]. A prospective study in China found that the velocity of BMI growth during young adulthood significantly affected the development of later-life diabetes [21]. Other prospective studies also suggested that early adulthood was of great importance for alleviating chronic disease burden across lifespan [22]. But, the metabolic diseases pattern or risk factors of younger adults were seldom investigated.

The disease burden of hypertension has been increasingly heavy worldwide and particularly in China [23]. Ethnic disparities in hypertension incidence have been observed by the Multi-Ethnic Study of Atherosclerosis (MESA), which demonstrated that the Black had significantly higher risk of developing hypertension compared with white people, but the hypertension incidence did not differ for Chinese and white participants [9]. However, this study only included middle-aged and older adults, and the hypertension burden of Chinese young adults and subjects of other races has never been compared. We found that the SBP and DBP level of Chinese was higher than that of other ethnic participants since early adulthood, and the prevalence of hypertension in Chinese young adults was the highest among the four populations. The prevalence of hypertension among U.S. adults of different ages reported by Nwankwo et al. was similar to our results [24]. Nevertheless, compared with other populations, much less hypertensive patients in China took anti-hypertensive medicine, especially the younger ones. The prevalence of undiagnosed hypertension and diabetes among Chinese over 45 years reported by Li and Lumey was 22.7 and 12.1% [25]. We reported higher prevalence of untreated hypertension and diabetes. It was possible that the people aware of their conditions could choose not to take anti-hypertensive or anti-diabetic medications, making the prevalence even higher. Early education and instruction of BP management are necessary for the young Chinese. What’s more, we noticed that the primary hypertension subtype in Chinese young subjects was SDH, while ISH or IDH was the major hypertension subtype in other racial/ethnic subjects. Previous studies observed that the CVD risk of SDH was significantly higher than the risk of IDH and ISH [26]. These results highlight the urgent need for management of hypertension in Chinese young adults to prevent CVD events in later life.

The prevalence of diabetes has also been escalating during the past decades. Previous researches indicated that compared with White people, East Asians were characterized primarily by β-cell dysfunction instead of insulin resistance [27, 28]. Nevertheless, a recent longitudinal study in China reported that insulin resistance showed a stronger association with incident diabetes than did β-cell dysfunction in Chinese adults [29]. We also demonstrated that although Chinese adults with diabetes were less characterized by insulin resistance than did other ethnic/racial populations, their insulin resistance pattern was still prominent, and this characteristic was more evident in obese subjects. Considering the increasing trend of obesity in China, it is essential to learn from the prevention strategies for diabetes based on mitigating insulin resistance in other racial/ethnic groups.

Hyperlipidemia is quite common among participants of different age. Increased LDL-C is closely associated with atherosclerosis, while the residual cardiovascular risk attributable to high TG has been attracting increasing attention [30]. We revealed that compared with Non-Hispanic Whites, the prevalence of hypertriglyceridemia was significantly higher, while that of high LDL-C was much lower in Chinese. Despite that the current guidelines for blood lipid management primarily recommend cholesterol-lowering therapy to reduce cardiovascular risk, [31] our results indicate that for Chinese adults, it is of great importance to manage triglyceride concentration besides LDL-C, to further reduce CVD risk associated with hyperlipidemia.

There is increasing evidence that patients with diabetes or hypertension diagnosed during young adulthood have a distinct cardiometabolic risk profile with older patients [32, 33]. A previous analysis of NHANES data showed that differences in severe obesity and lipid levels were more evident between young adults with and without diabetes compared with older adults with and without diabetes [34]. And obesity in young adults was reported to be associated with an increased prevalence of cardiometabolic risk factors [35]. We also found that in China, for risk of metabolic diseases, younger adults were more susceptible to obesity than older ones.. Besides, we noticed that BMI increased rapidly in young adulthood and peaked around 50 years. In the meantime, the pathophysiology of young subjects with diabetes was characterized by insulin resistance, and this pattern was more obvious in obese participants. Obesity also showed much stronger association with the risk of metabolic diseases in younger subjects than in the elders. Besides, physical inactivity which showed stronger association with diabetes in the young could increase metabolic risk by exacerbating obesity [36]. Considering the aggravating obesity epidemic in Chinese young adults, [37] it is fairly essential to encourage the young to exercise and keep in shape for prevention of metabolic disorders.

A major strength of present study is that the data are from participants in two nationwide surveys across China and United States, and it is representative of multiple ethnic populations over 18 years old. There are several limitations to consider in our study. First, the protocols of physical examinations and laboratory tests could be different among populations. And there was a potential bias due to the inconsistency of assessment tools. However, our major outcomes were hypertension, diabetes and hyperlipidemia, and the standards of measurement of blood pressure, glucose and lipids were majorly consistent among surveys in different populations. Questionnaires collecting information of risk factors were very different between the Chinese and NHANES surveys, so we performed risk factor analysis only in Mainland Chinese to avoid potential bias. Second, we assessed the pathophysiology of diabetes by surrogate markers, HOMA-IR and HOMA-B. It may lead to misclassification. However, HOMA-IR and HOMA-B are two classical markers used to classify diabetes subtypes in multiple studies [38, 39]. Third, we cannot infer causality due to the cross-sectional nature, further studies are needed to prospectively test the associations between metabolic risk factors and diseases in different age groups.

## Conclusions

The current research demonstrated that the prevalence, subtypes and risk factor profiles of metabolic diseases were distinct between young adults < 40 years and elder subjects. Young adults were more prone to obesity then the older ones. Besides, the metabolic diseases prevalence and subtype patterns showed evident disparities between Mainland Chinese and other ethnic/racial populations. People with different age or ethnicity/race should consider individualized management and prevention targets to reduce the risk of metabolic disorders.

## Supplementary Information

**Additional file 1: Supplementary Table 1.** Baseline characteristics of people of different ethnic and age. **Supplementary Table 2.** Age-specific odds ratios and young-to-old ratio of odds ratios for risk factors and metabolic diseases in the population including subjects on medication. **Supplementary Fig. 1.** Participant flow diagram of the subjects. **Supplementary Fig. 2.** Frequency distribution of untreated diabetic individuals by age and diabetes subtypes. **Supplementary Fig. 3.** Frequency distribution of untreated diabetic Chinese by age and diabetes subtypes in BMI subgroups. **Supplementary Fig. 4.** Frequency distribution of untreated diabetic individuals by age and diabetes subtypes. **Supplementary Fig. 5.** Association of age with cardiometabolic metrics in different racial/ethnic adults including those on medication.

## Data Availability

Data from the National Health and Nutrition Evaluation Survey can be downloaded from https://www.cdc.gov/nchs/nhanes/index.htm. Other data are available from the corresponding authors upon reasonable request.

## Abbreviations

2 h-PPG: 2 h post-load plasma glucose
ADA: American Diabetes Association
AHA: American Heart Association
BMI: Body mass index
CDC: Center for Disease Control
CI: Confidence interval
CVD: Cardiovascular diseases
DBP: Diastolic blood pressure
ESC: European Society of Cardiology
ESH: European Society of Hypertension
FPG: Fasting plasma glucose
HbA1c: Glycated hemoglobin
HOMA-IR: Homoeostasis model assessment of insulin resistance
HOMA-B: Homoeostasis model assessment of β-cell function
IDH: Isolated Diastolic Hypertension
ISH: Isolated Systolic Hypertension
LDL-C: Low-density lipoprotein cholesterol
MESA: Multi-Ethnic Study of Atherosclerosis
NCHS: National Center for Health Statistics
NHANES: National Health and Nutrition Evaluation Survey
OR: Odds ratio
ROR: Ratio of odds ratio
SBP: Systolic blood pressure
SDH: Systolic Diastolic Hypertension
TG: Triglycerides
U.S.: United States
WC: Waist circumstance
