# Supplementary material for: Age-specific prevalence, subtypes and risk factors of metabolic diseases in Chinese adults and the different patterns from other racial/ethnic populations
Source: BMC Public Health. 2022 Nov 14;22:2078. doi: 10.1186/s12889-022-14555-1 (PMC9664823; doi:10.1186/s12889-022-14555-1)

Supplemental material

Methods

Study population

For the current study, data of 98658 participants from the China Noncommunicable Disease Surveillance 2010 and 48527 individuals from the National Health and Nutrition Evaluation Survey (NHANES) 2005-2016 in races of Mexican American, Non-Hispanic White and Non-Hispanic Black was included. Individuals aged younger than 18 years (n=19445) and being pregnant at examination or uncertain of the pregnancy status (n=950) were excluded from the NHANES study. None of the participants had missing data in age. Among 126790 subjects involved in the analysis, 20,118 subjects were excluded (18,762 taking antihypertensive medicine and 1,356 missing data in blood pressure measurements), leaving 106,672 subjects included in the analysis of the associations between age and blood pressure, the hypertension subtypes and the risk factors for hypertension. Similarly, 25,713 subjects were excluded (6,533 taking antidiabetic medicine and 19,180 missing data in blood glucose measurements), leaving 101,077 subjects included in the analysis of the associations between age and blood glucose, the diabetes subtypes and the risk factors for diabetes. Similarly, 21,557 subjects were excluded (7,471 taking lipid lowering medicine and 14,086 missing data in blood lipids measurements), leaving 105,233 subjects included in the analysis of the associations between age and blood lipids, the hyperlipidemia subtypes and the risk factors for hyperlipidemia.

Data collection and risk factors definition

In China Noncommunicable Disease Surveillance 2010, trained staff obtained 3 blood pressure measurements from the nondominant arm of each participant with a 1-min interval by using a calibrated automatic electronic device (OMRON model HEM-7071, Omron Co., Kyoto, Japan) after at least a 5-min sitting rest. Before measurement, participants were advised to avoid alcohol, smoking, coffee, tea, and exercise at least 30 minutes. The average of 3 readings was used in the analysis. Blood samples were collected in all participants after an overnight fast of at least 10 hours. Serum lipids were measured by using an autoanalyzer (Abbott Laboratories). Fasting plasma glucose (FPG) and 2h post-load plasma glucose (2h-PPG) concentrations were measured locally using glucose oxidase or hexokinase methods within 24 hours.

General obesity was defined as a BMI of 25 kg/m^2 or greater. Central obesity was defined as waist circumstance of 90 cm or wider for men and 80 cm or wider for women. Higher education level was defined as achieving junior high school education or more. Lower family income was defined as family income less than 20,000 RMB/year. Lower occupation level was defined as support, service, unemployed or retiree. Unmarried or marital disruption (separation, divorce, being widowed) were defined as low grade marital status. Current smoking was defined as having smoked at least 100 cigarettes in life and smoking at present. Current drinking was defined as alcohol intake more than once per month during the past 12 months. The Global

Physical Activity Questionnaire was used to assess physical activity, and the metabolic equivalent (MET) was calculated to evaluate average weekly energy expenditure.[1] Physical inactivity was defined as 0 to <600 MET-min per week. A food frequency questionnaire of the previous 12 months was used to record habitual dietary intake. And the definition of unhealthy diet was having 0-1 component of healthy diet score, in accordance with a previous study.[2] The annual exposure to PM2.5 before the survey date at each participant's address was estimated using online data from the Chinese national urban air quality real-time release platform (https://air.cnemc.cn:18007/). Exposure to high PM2.5 level was defined as annual exposure to PM2.5 higher than the upper quartile.

Statistical analysis

To analyze metabolic diseases risk attributable to 11 modifiable risk factors (including general obesity, central obesity, lower education level, lower family income, lower occupation level, low grade marital status, current smoking, current drinking, physical inactivity, unhealthy diet and exposure to high PM2.5 level)in Mainland Chinese, we used 3 adjusted univariable logistic regression models to identify the age-specific odds ratios and 95%CI for each risk factor for metabolic diseases. The model used for hypertensive risk factors analysis was adjusted for age (as a continuous variable), sex, hypertension family history and diagnosis of diabetes and hyperlipidemia. The model used for diabetic risk profiles analysis was adjusted for age, sex, diabetes family history and diagnosis of hypertension and hyperlipidemia. The model used for hyperlipidemic risk profiles analysis was further adjusted for age, sex and diagnosis of hypertension and diabetes.

References

1. Craig CL, Marshall AL, Sjöström M, et al. International physical activity questionnaire: 12-country reliability and validity. *Med Sci Sports Exerc* 2003;35(8):1381-95.
2. Bi Y, Jiang Y, He J, et al. Status of cardiovascular health in Chinese adults. *J Am Coll Cardiol* 2015;65(10):1013-25. doi: 10.1016/j.jacc.2014.12.044 [published Online First: 2015/03/15]

Supplementary Table 1. Baseline characteristics of people of different ethnic and age

Characteristics	Mainland Chinese		Mexican American		Non-Hispanic White		Non-Hispanic Black	
	< 40 years	≥40 years	< 40 years	≥40 years	< 40 years	≥40 years	< 40 years	≥40 years
Number	32721	65937	2493	3233	4675	10057	2857	4817
Age, years (95%CI)	28.5 (28.2-28.8)	54.7 (54.3-55.1)	28.5 (28.2-28.8)	53.5 (52.8-54.1)	28.5 (28.2-28.8)	58.7 (58.2-59.1)	27.9 (27.5-28.3)	56.0 (55.6-56.5)
Male, % (95%CI)	51.5 (50.6-52.4)	50.2 (49.2-51.1)	57.7 (56.0-59.4)	50.5 (48.8-52.2)	53.4 (51.9-54.9)	47.8 (46.9-48.7)	49.0 (47.0-51.0)	44.0 (43.0-45.0)
BMI, kg/m^2 (95%CI)	23.1 (22.9-23.3)	24.2 (24.1-24.4)	29.2 (28.8-29.5)	30.4 (30.1-30.6)	27.5 (27.2-27.8)	29.1 (28.9-29.3)	29.8 (29.4-30.1)	31.1 (30.8-31.4)
WC, cm (95%CI)	78.0 (77.4-78.6)	82.1 (81.5-82.7)	96.8 (95.9-97.6)	101.7 (101.1-102.4)	93.7 (92.9-94.5)	101.6 (101.1-102.0)	95.2 (94.4-96.1)	102.5 (101.9-103.2)
SBP, mmHg (95%CI)	122.7 (121.7-123.7)	139.3 (138.2-140.4)	115.3 (114.7-115.9)	126.0 (125.2-126.9)	114.7 (114.2-115.2)	125.6 (125.1-126.2)	118.1 (117.4-118.9)	131.3 (130.5-132.1)
DBP, mmHg (95%CI)	77.3 (76.7-77.8)	83.8 (83.3-84.3)	67.3 (66.7-68.0)	71.6 (71.0-72.2)	68.7 (68.2-69.3)	70.9 (70.5-71.4)	68.7 (67.9-69.5)	73.0 (72.3-73.7)
HbA1c, % (95%CI)	5.60 (5057-5.62)	5.93 (5.90-5.96)	5.41 (5.36-5.45)	6.13 (6.05-6.21)	5.19 (5.17-5.21)	5.69 (5.67-5.72)	5.47 (5.43-5.51)	6.08 (6.04-6.12)
FPG, mg/dl (95%CI)	95.9 (94.6-97.1)	104.3 (103.1-105.6)	101.7 (99.8-103.5)	119.9 (117.0-122.8)	96.5 (95.7-97.3)	109.1 (107.9-110.2)	97.3 (95.6-99.0)	114.6 (112.7-116.5)
2h-PPG, mg/dl (95%CI)	103.3 (101.4-105.3)	120.2 (118.3-122.0)	109.9 (105.9-113.9)	135.9 (131.9-140.0)	98.1 (96.5-99.6)	124.0 (121.7-126.4)	99.3 (96.8-101.7)	123.5 (120.4-126.7)
HOMA-IR (95%CI)	1.71 (1.65-	1.68 (1.62-	3.24 (2.93-	4.65 (4.14-	2.75 (2.38-	3.89 (3.54-	3.24 (2.58-	3.80 (3.36-

	1.77)	1.74)	3.54)	5.16)	3.12)	4.23)	3.90)	4.24)
**HOMA-B (95%CI)**	85.4 (81.2-89.7)	63.5 (61.0-66.1)	121.9 (115.5-128.4)	108.3 (102.7-114.0)	112.3 (104.1-120.6)	96.5 (92.7-100.3)	132.3 (121.5-143.1)	106.2 (100.3-112.1)
**Total cholesterol, mg/dl (95%CI)**	147.9 (145.1-150.6)	165.7 (162.8-168.6)	186.3 (184.2-188.4)	200.7 (198.9-202.5)	182.9 (181.5-184.3)	201.1 (199.9-202.3)	176.7 (175.1-178.2)	194.8 (193.2-196.4)
**Triglyceride, mg/dl (95%CI)**	111.3 (107.5-115.0)	131.4 (128.0-134.8)	129.3 (121.9-136.7)	150.7 (143.6-157.8)	114.2 (109.6-118.8)	136.5 (132.8-140.1)	88.5 (82.7-94.3)	105.2 (100.8-109.7)
**LDL-C, mg/dl (95%CI)**	82.4 (80.6-84.2)	93.7 (91.8-95.7)	109.6 (106.9-112.2)	119.3 (117.2-121.4)	106.8 (105.2-108.3)	117.5 (116.2-118.8)	105.1 (103.0-107.1)	115.6 (113.5-117.7)
**HDL-C, mg/dl (95%CI)**	41.9 (41.0-42.7)	43.4 (42.7-44.2)	48.5 (47.8-49.3)	49.9 (49.1-50.6)	51.7 (51.0-52.3)	54.8 (54.3-55.4)	54.0 (53.4-54.7)	57.4 (56.9-58.0)

CI, confidence interval; BMI, body mass index; WC, waist circumstance; SBP, systolic blood pressure; DBP, diastolic blood pressure; HbA1c, glycated hemoglobin; FPG, fasting plasma glucose; 2h-PPG, 2h-postload plasma glucose; HOMA-IR, homoeostasis model assessment of insulin resistance index; HOMA-B, homoeostasis model assessment of β-cell function index; LDL-C, low density lipoprotein cholesterol; HDL-C, high density lipoprotein cholesterol.

Supplementary Table 2. Age-specific odds ratios and young-to-old ratio of odds ratios for risk factors and metabolic diseases in the population including subjects on medication

Hypertension

Risk factors	OR (95%CI)	P for interaction	Young: Old ROR (95%CI)
**General obesity**			
18-40 years	2.72 (2.51-2.95)	<0.001	1.35 (1.23-1.47)
>40 years	2.07 (1.98-2.17)		
**Central obesity**			
18-40 years	3.88 (3.21-4.68)	<0.001	1.38 (1.16-1.64)
>40 years	2.34 (2.12-2.57)		
**Education less than high school**			
18-40 years	1.14 (0.99-1.32)	0.147	0.90 (0.77-1.04)
>40 years	1.14 (1.05-1.24)		
**Lower family income**			
18-40 years	1.02 (0.87-1.20)	0.008	0.83 (0.72-0.95)
>40 years	1.20 (1.10-1.30)		
**Lower occupation level**			
18-40 years	0.96 (0.82-1.11)	0.948	1.00 (0.87-1.16)
>40 years	0.87 (0.79-0.96)		
**Low grade marital status**			
18-40 years	0.98 (0.85-1.13)	0.962	1.00 (0.91-1.09)
>40 years	0.99 (0.90-1.08)		
**Current smoking**			
18-40 years	0.79 (0.70-0.89)	<0.001	1.34 (1.20-1.50)
>40 years	0.86 (0.81-0.92)		
**Current drinking**			
18-40 years	1.11 (0.97-1.27)	0.003	1.19 (1.06-1.33)
>40 years	1.29 (1.21-1.38)		
**Physical inactivity**			
18-40 years	1.01 (0.88-1.16)	0.602	1.04 (0.94-1.12)
>40 years	1.05 (0.96-1.15)		
**Unhealthy diet**			
18-40 years	1.06 (0.92-1.21)	0.707	0.97 (0.85-1.11)
>40 years	1.08 (1.00-1.17)		
**Exposure to high level of PM2.5**			
18-40 years	1.19 (0.93-1.51)	0.370	0.92 (0.77-1.11)
>40 years	1.29 (1.09-1.52)		

Diabetes

Risk factors	OR (95%CI)	P for interaction	Young: Old ROR (95%CI)
**General obesity**			
18-40 years	1.72 (1.51-1.96)	0.029	1.15 (1.01-1.31)

>40 years	1.58 (1.49-1.69)		
**Central obesity**			
18-40 years	3.20 (2.43-4.22)	<0.001	1.68 (1.31-2.15)
>40 years	1.98 (1.79-2.18)		
**Education less than high school**			
18-40 years	0.95 (0.79-1.13)	0.866	1.02 (0.82-1.26)
>40 years	0.89 (0.81-0.98)		
**Lower family income**			
18-40 years	1.12 (0.96-1.30)	0.253	1.11 (0.93-1.32)
>40 years	1.00 (0.91-1.10)		
**Lower occupation level**			
18-40 years	1.13 (0.88-1.44)	0.503	0.91 (0.70-1.19)
>40 years	1.20 (1.06-1.37)		
**Low grade marital status**			
18-40 years	0.97 (0.76-1.23)	0.702	1.04 (0.84-1.30)
>40 years	0.95 (0.86-1.05)		
**Current smoking**			
18-40 years	0.77 (0.63-0.94)	0.365	0.92 (0.78-1.10)
>40 years	0.95 (0.86-1.04)		
**Current drinking**			
18-40 years	0.76 (0.62-0.92)	0.080	0.84 (0.69-1.02)
>40 years	1.05 (0.96-1.15)		
**Physical inactivity**			
18-40 years	1.34 (1.11-1.62)	0.003	1.36 (1.11-1.65)
>40 years	1.00 (0.94-1.08)		
**Unhealthy diet**			
18-40 years	0.98 (0.82-1.16)	0.896	1.01 (0.83-1.23)
>40 years	0.97 (0.88-1.07)		
**Exposure to high level of PM2.5**			
18-40 years	1.04 (0.77-1.40)	0.532	1.11 (0.79-1.56)
>40 years	0.95 (0.78-1.16)		

Dyslipidemia

**Risk factors**	**OR (95%CI)**	**P for interaction**	**Young: Old ROR (95%CI)**
**General obesity**			
18-40 years	2.60 (2.40-2.80)	<0.001	1.35 (1.24-1.46)
>40 years	1.99 (1.89-2.08)		
**Central obesity**			
18-40 years	2.96 (2.54-3.44)	0.014	1.22 (1.04-1.43)
>40 years	2.02 (1.88-2.18)		
**Education less than high school**			
18-40 years	0.91 (0.81-1.02)	0.001	1.24 (1.09-1.41)
>40 years	0.80 (0.74-0.86)		

**Lower family income**			
18-40 years	0.97 (0.86-1.11)	<0.001	1.24 (1.11-1.39)
>40 years	0.80 (0.75-0.85)		
**Lower occupation level**			
18-40 years	0.90 (0.79-1.03)	<0.001	0.65 (0.55-0.76)
>40 years	1.16 (1.06-1.28)		
**Low grade marital status**			
18-40 years	0.87 (0.75-1.00)	<0.001	0.69 (0.59-0.80)
>40 years	1.01 (0.94-1.10)		
**Current smoking**			
18-40 years	1.28 (1.15-1.43)	<0.001	1.78 (1.59-1.98)
>40 years	1.07 (1.00-1.16)		
**Current drinking**			
18-40 years	1.31 (1.19-1.44)	<0.001	1.63 (1.48-1.80)
>40 years	1.15 (1.09-1.23)		
**Physical inactivity**			
18-40 years	1.25 (1.13-1.39)	0.159	1.09 (0.97-1.23)
>40 years	1.20 (1.11-1.31)		
**Unhealthy diet**			
18-40 years	1.01 (0.92-1.11)	0.129	1.08 (0.98-1.20)
>40 years	0.93 (0.86-0.99)		
**Exposure to high level of PM2.5**			
18-40 years	0.91 (0.75-1.10)	0.827	0.98 (0.85-1.13)
>40 years	0.91 (0.79-1.05)		

Supplementary Figure 1. Participant flow diagram of the subjects

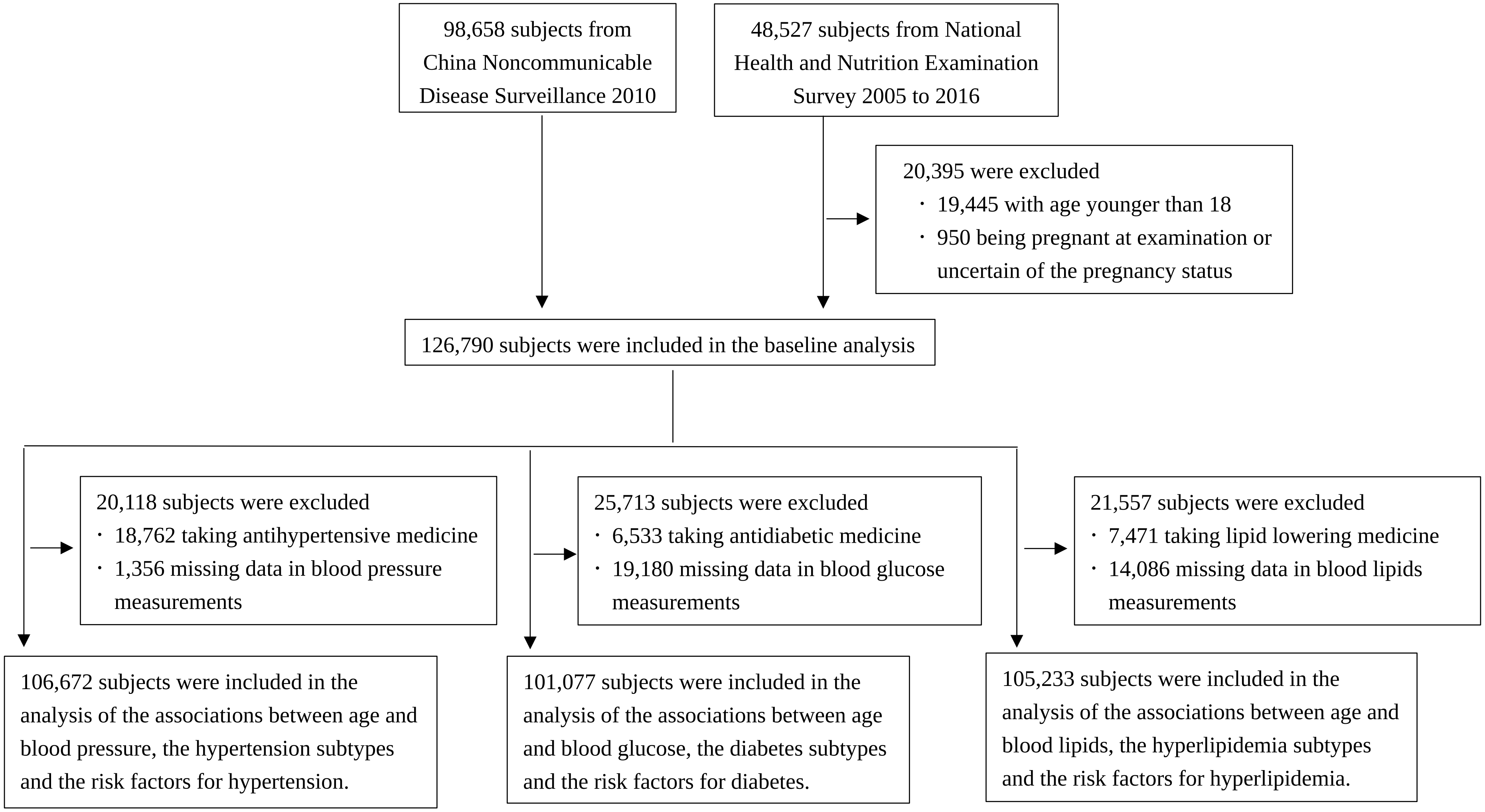

Supplementary Figure 2. Frequency distribution of untreated diabetic individuals by age and diabetes subtypes.

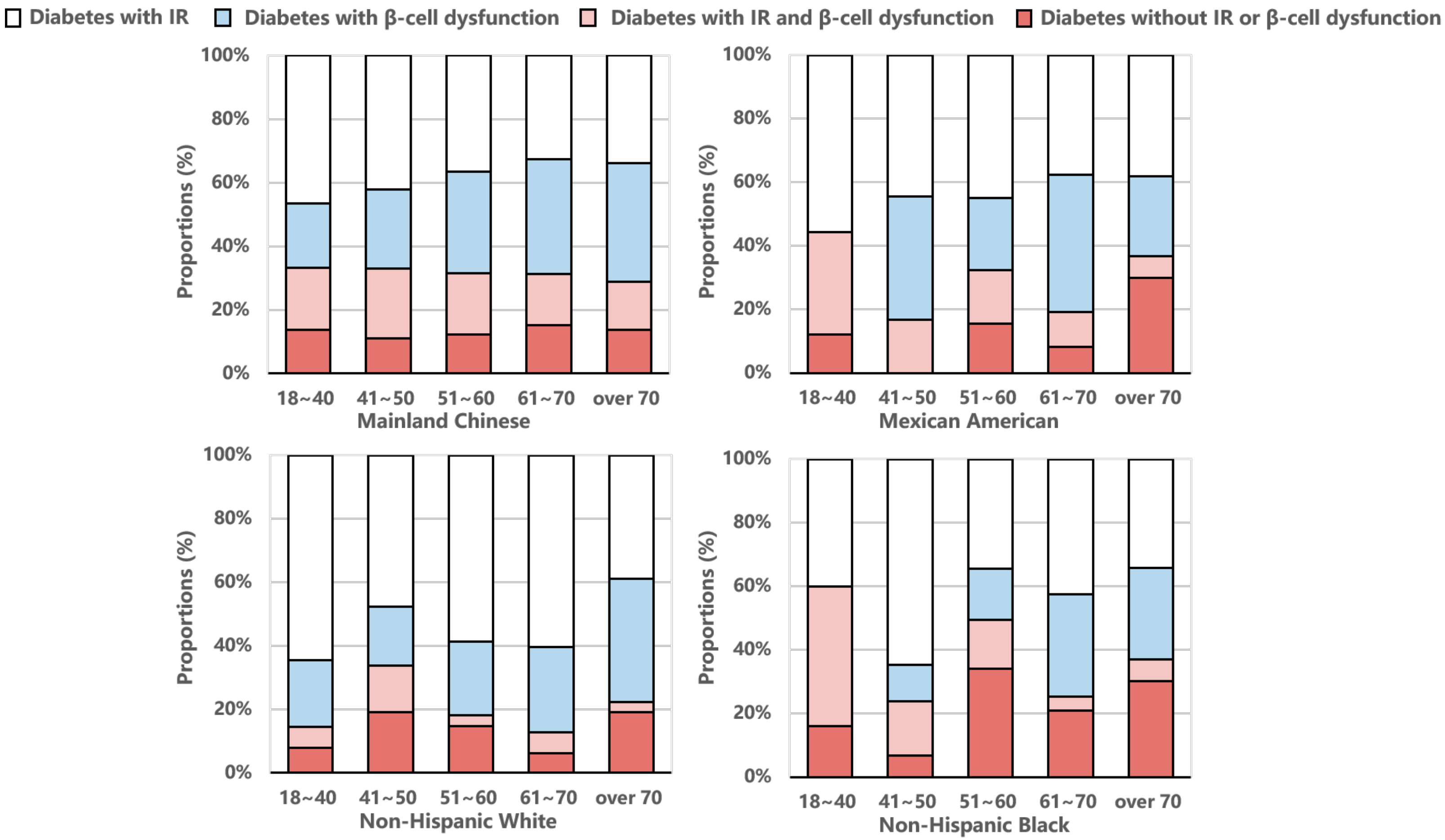

IR, insulin resistance.

Supplementary Figure 3. Frequency distribution of untreated diabetic Chinese by age and diabetes subtypes in BMI subgroups.

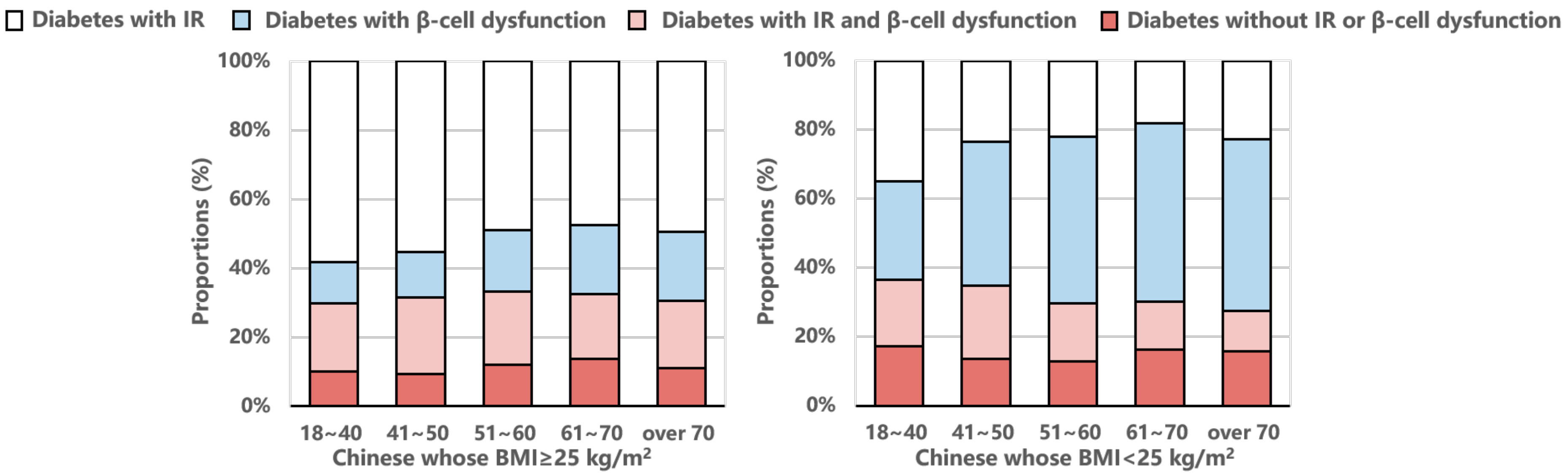

IR, insulin resistance; BMI, body mass index.

Supplementary Figure 4. Frequency distribution of untreated diabetic individuals by age and diabetes subtypes.

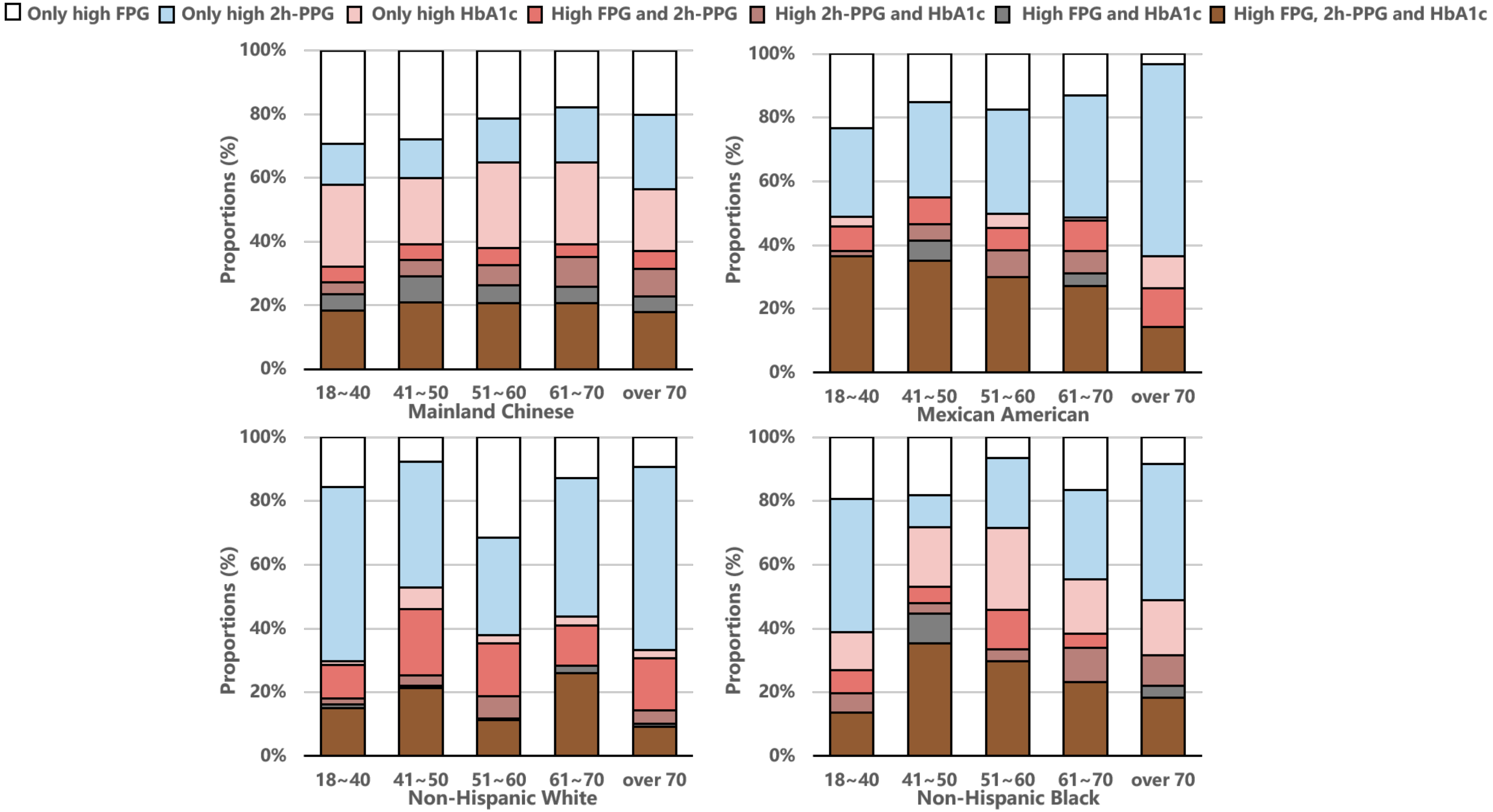

FPG, fasting plasma glucose; 2h-PPG, 2h-postload plasma glucose; HbA1c, glycated hemoglobin.

Supplementary Figure 5. Association of age with cardiometabolic metrics in different racial/ethnic adults including those on medication

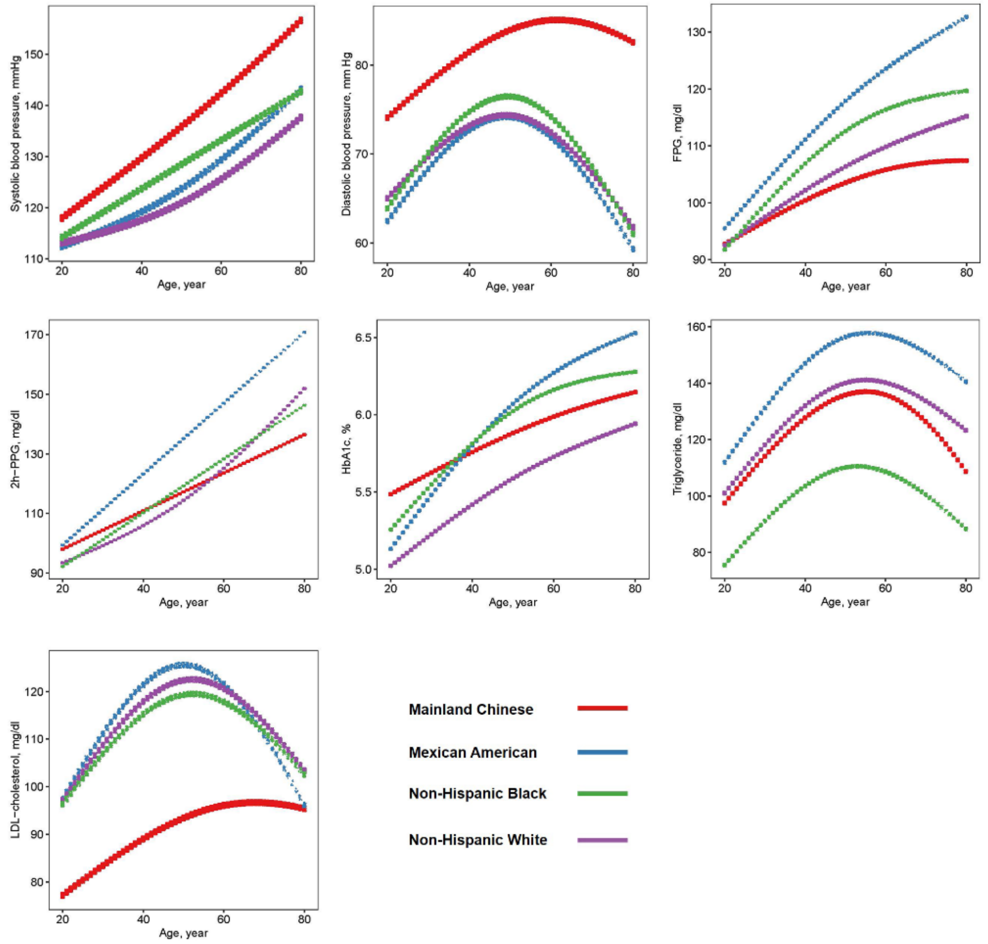

Supplementary Figure 6. Frequency distribution of untreated hypertensive individuals by age and hypertension subtypes including those under anti-hypertensive treatment

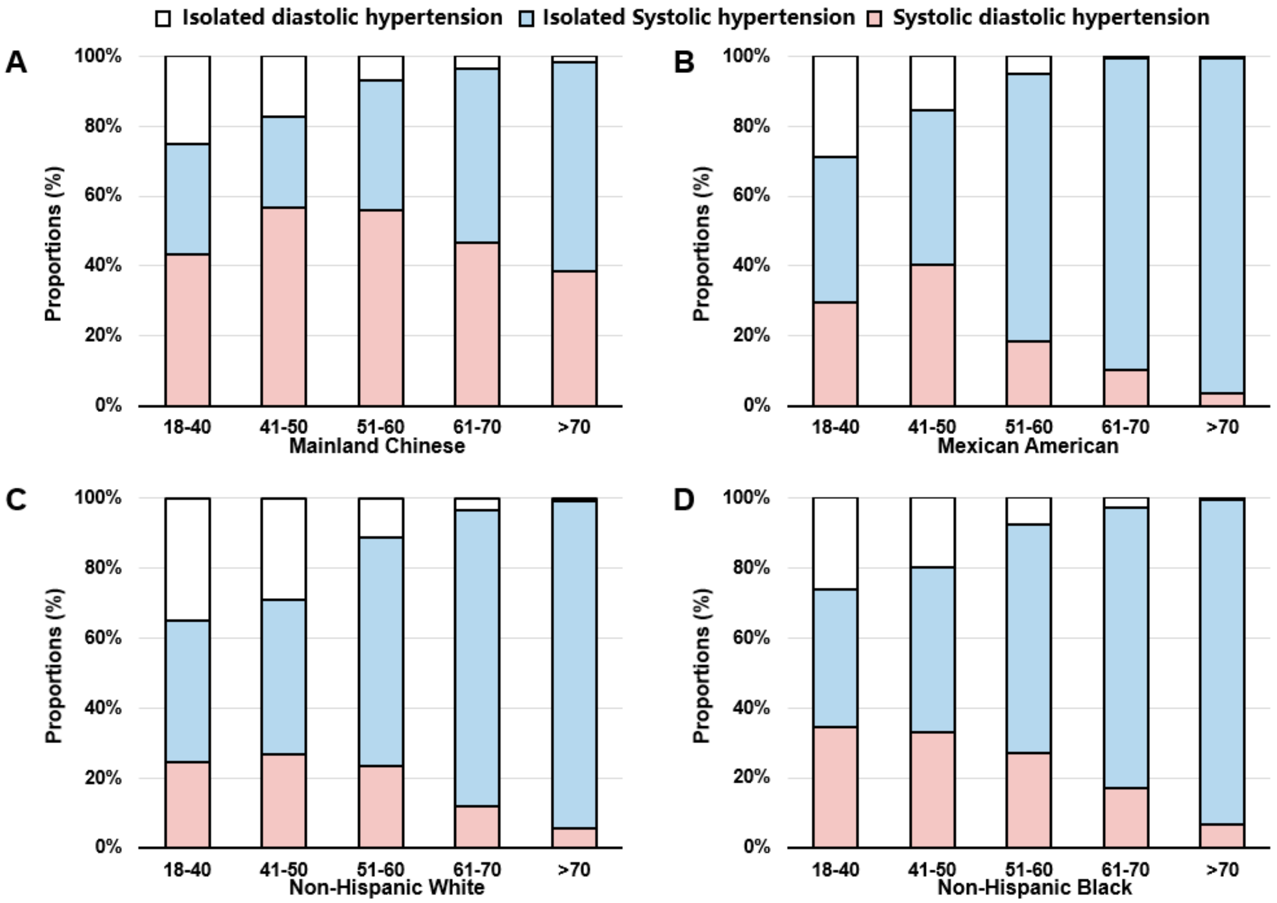

Supplement: Supplementary file 1 — Additional file 1: Supplementary Table 1. Baseline characteristics of people of different ethnic and age. Supplementary Table 2. Age-specific odds ratios and young-to-old ratio of odds ratios for risk factors and metabolic diseases in the population including subjects on medication. Supplementary Fig. 1. Participant flow diagram of the subjects. Supplementary Fig. 2. Frequency distribution of untreated diabetic individuals by age and diabetes subtypes. Supplementary Fig. 3. Frequency distribution of untreated diabetic Chinese by age and diabetes subtypes in BMI subgroups. Supplementary Fig. 4. Frequency distribution of untreated diabetic individuals by age and diabetes subtypes. Supplementary Fig. 5. Association of age with cardiometabolic metrics in different racial/ethnic adults including those on medication. [file 12889_2022_14555_MOESM1_ESM.pdf]
